# Educational intervention on sleep hygiene habits and sleep quality of postgraduate students^1^

**DOI:** 10.1590/1518-8345.7996.4765

**Published:** 2026-03-16

**Authors:** Ariane Naidon Cattani, Carmem Lúcia Colomé Beck, Rosângela Marion da Silva, Sandra Soares Mendes, Regina Célia Gollner Zeitoune, Silviamar Camponogara

**Affiliations:** 1 Universidade Federal de Santa Maria, Santa Maria, RS, Brazil.; 2 Scholarship holder at the Conselho Nacional de Desenvolvimento Científico e Tecnológico (CNPq), Brazil.; 3 Centro Universitário das Faculdades Associadas de Ensino, Departamento de Enfermagem, São João da Boa Vista, SP, Brazil.; 4 Universidade Federal do Rio de Janeiro, Rio de Janeiro, RJ, Brazil.

**Keywords:** Employee Health, Sleep Hygiene, Sleep Quality, Sleep, Health Postgraduate Programs, Nursing.

## Abstract

**Objective:**

to analyze the effect of an educational intervention on the sleep hygiene habits and sleep quality of *stricto sensu* graduate nursing students.

**Method:**

a quasi-experimental, before-and-after study with students from a graduate nursing program was conducted. A socio-occupational questionnaire, lifestyle habits questionnaire, sleep habits questionnaire, a questionnaire to assess the ease or difficulty of implementing the strategies, the Pittsburgh Sleep Quality Index and the Sleep Hygiene Index were used. The intervention consisted of an online session with guidance on sleep hygiene habits, a handout and video with the content were presented and text messages to reinforce the guidance. The McNemar test was used for pre- and post-intervention comparisons with a 5% significance level (p<0.05).

**Results:**

a total of 40 graduate students participated in the study. Sleep hygiene improved after the intervention (p=0.012). Participants increased their sleep hours (p=0.049), perceived improvement in sleep (p=0.016), acquired sleep hygiene habits (p<0.001) and there was a reduction in the number of students who woke up feeling tired (p=0.039).

**Conclusion:**

the intervention improved sleep hygiene and the subjective perception of sleep among postgraduate students.

## Highlights

**(1)** The intervention performed improved sleep hygiene, subjective perception, and total sleep time. **(2)** The intervention resulted in improvements in sleep habits and perception of fatigue. **(3)** Sleep hygiene is an accessible practice that enables self-care.

## Introduction

Sleep is a basic human need which promotes well-being, physical and mental health when adequate. Despite being essential for human beings, it is estimated that 20 to 45% of the world’s population is sleep deprived^1^, which represents a growing challenge for public health.

Biological and environmental factors, combined with inappropriate behaviors and habits, can lead to insufficient rest and excessive daytime sleepiness, as well as impairments in people’s daily lives, as they interfere with performance and quality of life^2^. These situations can have significant social and economic impacts, including increased use of medical resources and pharmaceutical services, as well as expenses related to absenteeism, reduced academic and work productivity and increased workplace accidents^3^.

Graduate nursing students, like those in other graduate programs, face high academic, professional and social demands, in addition to facing difficulties related to mental distress, lack of financial support for research and an accumulation of employment relationships alongside course activities^4^. It is important to note that these professionals are mostly nurses who are exposed to a complex work environment and must deal with caring for the health and lives of others. This requires attention, concentration, quick decision-making and the need for continuous study and updating, which demands time and reduces rest hours^3^.

This situation can be exacerbated by poor daily habits, such as excessive and inappropriate intake of stimulants, excessive use of screens like smartphones, computers and tablets and studying, working and performing activities that require high brain activity in bed or before bed. Sleep deprivation and difficulty in maintaining a proper routine impair cognitive performance, memory, concentration and the cardiovascular, metabolic, immune and hormonal systems, favoring anxiety and depression symptoms with detrimental effects on emotional health^1^.

Given these facts, the importance of implementing interventions aimed at improving sleep and health through self-care is emphasized. Sleep hygiene practices used in other studies which have shown improvements in overall sleep are suggested^2^ ^,^ ^5^ ^-^ ^6^. This practice refers to adopting basic habits that can support a healthier sleep pattern. It can be guided through an educational intervention which aims to explain behavioral and stimulus control techniques to reduce factors which are detrimental to sleep, including sleep latency, duration, quality and subjective perception^7^. It is suggested that guidance be provided verbally and in writing^8^.

Habits include maintaining a regular bedtime and wake-up time, avoiding stimulant consumption before bed, exercising regularly and maintaining a pleasant environment for rest^8^ ^-^ ^9^. An educational intervention on sleep hygiene is one way to educate people about self-care^10^.

Thus, an integrative review was conducted in 2024 as part of a graduate course to deepen knowledge about interventions to improve sleep. No studies involving graduate students were identified, highlighting a gap in knowledge production and highlighting the need for intervention studies targeting this population.

In turn, we sought to elucidate the research question: does an educational intervention on sleep hygiene improve the sleep habits and quality of sleep among *stricto sensu* graduate nursing students? The objective of this study was therefore to analyze the effect of an educational intervention on the sleep hygiene habits and quality of sleep among *stricto sensu* graduate nursing students.

## Method

### Study design

This was a quasi-experimental, non-randomized before-and-after study that included an online educational intervention on sleep hygiene habits. The study followed the guidelines of the Strengthening the Reporting of Observational Studies in Epidemiology (Standards for Quality Improvement Reporting Excellence) (SQUIRE 2.0) checklist.

### Study location

The study was conducted in a *stricto sensu* Graduate Program in Nursing (PPGEnf) at a Federal University in Rio Grande do Sul, Brazil. The master’s program had a total workload of 360 hours, while the doctorate program totaled 600 hours. Students completed required and optional courses, prepared and defended their dissertations or theses and participated in research group activities, the program and scientific events. Admissions were conducted annually by advisor and an average of 22 places were offered for each program.

### Study population and sample

The study was conducted with master’s and doctorate students of the *stricto sensu* Graduate Program in Nursing (PPGEnf), Brazil. A total of 133 students were enrolled during the data collection period, composed of 64 master’s students and 69 doctoral students. Convenience (non-probabilistic) sampling was chosen due to participant availability. Data collection took place from May 2023 to February 2024. 

### Selection criteria

The inclusion criteria were being between 18 and 59 years of age (due to the particularities related to sleep in each age group) and being regularly enrolled in the PPGEnf program; the exclusion criteria were being on leave or any type of absence during the data collection period. It is worth clarifying that participants were not asked prior to receiving the data collection instruments whether they were using medications with effects on sleep, apnea/hypopnea, neuropsychiatric disorders or chronic pain. 

### Educational intervention

The educational intervention consisted of an online session in which one of the authors, a graduate student with experience in the topic and quantitative data collection, provided verbal guidance through a dialogued presentation on general aspects, phases and importance of sleep, circadian rhythm and potential harm to physical, mental and emotional health resulting from sleep disturbances. She also discussed sleep hygiene habits, introducing the concept, personal behaviors and environmental factors that can help improve sleep. Participants were invited to implement these habits into their daily routines.

The content covered in the educational intervention was based on scientific evidence identified in a previously conducted integrative review and the guidelines of the Brazilian Sleep Association^8^ ^-^ ^9^, adapted for the study population. These guidelines included: going to bed at night when drowsy; maintaining a regular bedtime and wake-up routine (regardless of bedtime), including on weekends; avoiding staying in bed for more than 30 minutes while awake; if you need to get up, avoid white or blue light (find a place to relax outside the bedroom and only return to bed when you are sleepy, even during the early hours of the morning); avoid using your bed for tasks that stimulate brain activity (reading, working, watching TV, using your cell phone, or any other brightly-emitting screen); avoid stressful activities before bed, writing down your appointments in your calendar and setting aside time during the day to focus on your concerns; avoid napping during the day (if necessary, limit the duration to approximately 30 minutes after lunch); limit the use of alcohol, nicotine and stimulants (coffee, mate tea, energy drinks) after 3 p.m.; avoid overeating approximately four hours before bed; exercise regularly (up to one hour before bedtime); take a warm bath close to bedtime to relax; practice breathing control exercises at any time of the day to relax and keep your sleeping environment dark, quiet and at a comfortable temperature.

The online sessions took place in different shifts and a total of 21 sessions were held via Google Meet. Each participant was assigned to a group to participate in the session, according to the previously announced date and time. Groups were formed to allow for discussion. There were five groups in the morning session, 12 groups in the afternoon session, and four groups in the evening session. Groups had a minimum of one and a maximum of seven participants and each individual participated once. Despite the variable number of participants in each meeting, the intervention strictly followed a previously prepared script. 

Each meeting lasted approximately 25-30 minutes. Approximately 10 minutes were allocated at the end of each session for participants to share their experiences, challenges and strengths related to their routines and sleep, as well as to clarify any questions about the topic.

Then, a Portable Document Format (PDF) brochure and a 1-minute and 20-second video, both created by the authors, with sleep hygiene guidelines were sent via WhatsApp^®^ to each participant present immediately after the end of each online session, allowing access to the content whenever desired.

Finally, four text messages were sent, one per week, encouraging and reminding participants about their habits, providing an opportunity to clarify any questions and share their experiences. The first message was sent seven days after participation in the educational intervention; the second message, seven days after the first and so on.

### Instruments

A socio-occupational questionnaire was used with a quantitative variable related to age and qualitative variables, categorized as yes or no, related to sex assigned at birth, children, graduate school, commuting to graduate school and work. A lifestyle questionnaire with qualitative variables (also categorized as yes or no) related to the use of sleeping medications and self-reported health symptoms was also used. A sleep habits questionnaire with variables on duration, latency, perception of sleep, sleepiness, sleep hygiene habits and bedtime routine, was additionally used.

Participants were asked to select the option “easy” or “difficult” for questions to assess the degree of ease or difficulty in falling asleep, such as: “What was it like for you at night going to bed when you were sleepy? What was it like for you to maintain a regular bedtime and wake-up routine?” These questions were developed by a doctoral student and analyzed by two professors with doctoral degrees and experienced in research on workers’ health and sleep.

The Pittsburgh Sleep Quality Index, a validated version in Brazilian Portuguese (PSQI-BR), was used to assess sleep quality. Its overall score can range from 0 to 21 points, classifying sleep quality as good (≤ 4), poor sleep quality (5-10), and presence of sleep disorder (≥ 11)^11^. It was decided to categorize the variable as “good” (≤ 5) and “poor” (> 5 points) sleep quality, as used in another study^12^.

Next, the Sleep Hygiene Index, a validated version in Brazilian Portuguese (IHS-BR), was used to measure sleep hygiene habits. Its score can range from 13 to 65 points, with higher scores indicating inadequate sleep hygiene^13^. Sleep hygiene for this study was considered “average/good” (≤34 points) and “poor” (≥35 points)^14^ ^-^ ^15^. 

The data collection instruments were pilot-tested with participants in a research group to assess question clarity, completion time and potential interpretation difficulties. After formatting adjustments, the instruments were deemed suitable for data collection. 

### Data collection

**Figure 1:**
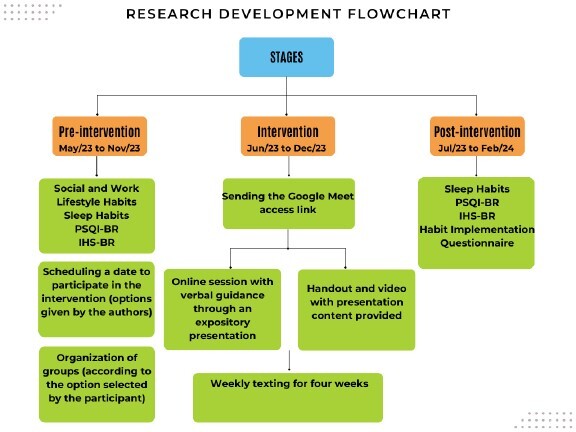
Research development flowchart

Data collection took place online using Google Forms in three stages: before (pre-intervention), during implementation and after the intervention (post-intervention). The intervention was offered after completing the pre-intervention questionnaires and consisted of a dialogued presentation and four text messages to reinforce sleep hygiene habits. The post-intervention questionnaires were made available one week after the fourth and final message was sent. A flowchart is presented in Figure 1 to better visualize the study development stages.

### Data analysis

The collected data were exported from Google Forms, organized in Microsoft Excel^®^ software and analyzed in the Statistical Package for the Social Sciences^®^ (SPSS 25.0). Categorical variables are described by absolute (N) and relative (%) frequency and quantitative variables by mean and standard deviation. The change between time points for each sleep habit, sleep quality and sleep hygiene index was described and compared before and after the intervention using McNemar’s chi-squared test, considering a significance level of 5% (p<0.05) to reject the null hypothesis.

### Ethical aspects

Ethical aspects were respected, as recommended by Resolutions 466/12 and 510/2016 of the National Health Council. The study was approved by the Research Ethics Committee of the Federal University of Santa Maria, with Certificate of Presentation of Ethical Appreciation No. 66359622.6.0000.5346, under Opinion No. 5.847.306 of January 2023. It is mentioned that the Informed Consent Form included information about the use of text messages via WhatsApp^®^.

## Results

A total of 53 nursing graduate students (39.84% of the population) completed the pre-intervention questionnaires; of these, 11 did not participate in the intervention and two did not respond to the post-intervention questionnaires. Thus, 40 participants completed all phases of the study and were considered for comparative analysis. The participants had a mean age of 34 years (standard deviation ± 8.11) (minimum, 23; maximum, 54 years), 95% (n = 38) self-identified as female and 42.5% (n = 17) had children. Of the participants, 50% (n = 20) were pursuing a doctorate, 25% (n = 10) needed to travel to another city or state to complete their postgraduate studies, 87.5% (n = 35) worked, 37.5% (n = 15) were undergoing some form of health treatment and 20% (n = 8) used sleeping medication. Analyses were conducted before and after the intervention (n=40) comparing participants’ sleep habits, sleep quality (PSQI-BR) and sleep hygiene (IHS-BR). Table 1 below represents the pre- and post-intervention comparison of variables related to the sleep habits of graduate students.

**Table 1:** Variables related to sleep habits before and after the intervention with postgraduate students (n = 40). Santa Maria, RS, Brazil, 2023-2024

Variable	Before the intervention n (%)	After the intervention n (%)	p-value*
Consider the hours of sleep sufficient			
Yes	16 (40.0)	24 (60.0)	0.057
No	24 (60.0)	16 (40.0)	
Difficulty staying asleep			
Yes	15 (37.5)	9 (22.5)	0.146
No	25 (62.5)	31 (77.5)	
Difficulty initiating sleep			
Yes	13 (32.5)	8 (20.0)	0.125
No	27 (67.5)	32 (80.0)	
Difficulty waking up			
Yes	23 (57.5)	16 (40.0)	0.065
No	17 (42.5)	24 (60.0)	
Daytime sleepiness			
Yes	26 (65.0)	22 (55.0)	0.388
No	14 (35.0)	18 (45.0)	
Total	40 (100)	40 (100)	

*McNemar chi-squared test, significant if p-value < 0.05

**Figure 2:**
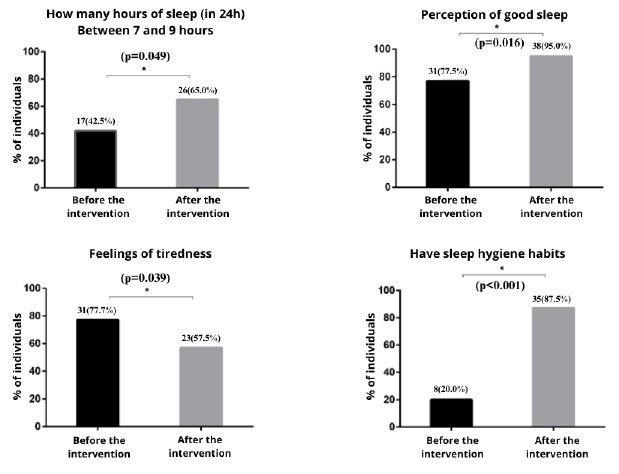
Sleep habits before and after the intervention with postgraduate students (n = 40). Santa Maria, RS, Brazil, 2023-2024

No statistical significance was found in these sleep habits. Figure 2 below shows the statistical difference between the number of hours of sleep, perception of sleep, feelings of tiredness and sleep hygiene habits before and after the intervention.

*McNemar chi-squared test, significant if p-value < 0.05

**Figure 3:**
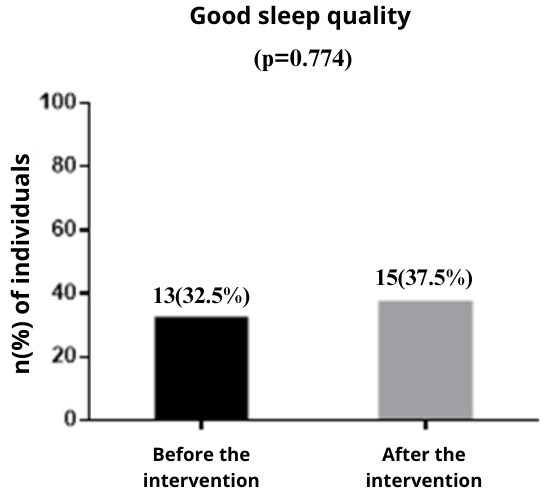
Sleep quality before and after intervention with postgraduate students (n = 40). Santa Maria, RS, Brazil, 2023-2024

There was a percentage increase in participants who increased their sleep hours after the intervention from <7h to ≥7h to 9h per night (p=0.049) (22.5%), who perceived improvement in their sleep (p=0.016) (17.5%) and who acquired sleep hygiene habits (p<0.001) (67.5%). There was a percentage reduction in students who woke up feeling tired (p=0.039) (20.2%). Figure 3 shows the quality of sleep according to the PSQI-BR before and after the intervention.

*McNemar chi-squared test, significant if p-value < 0.05

**Figure 4:**
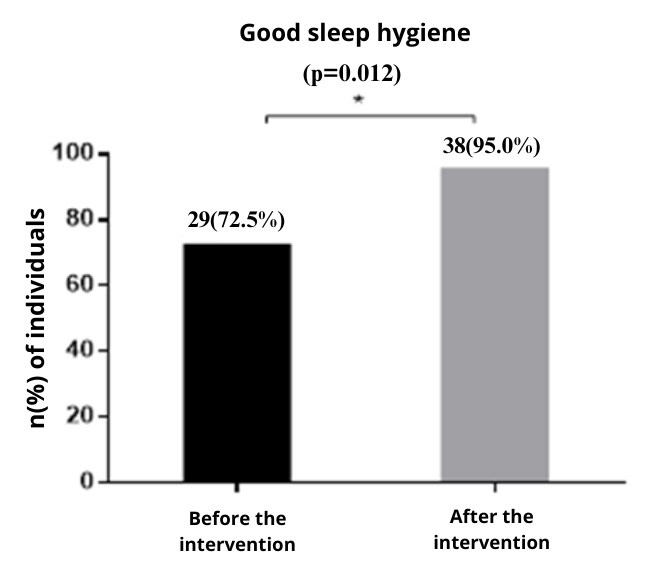
Sleep hygiene before and after intervention with postgraduate students (n = 40). Santa Maria, RS, Brazil, 2023-2024

Sleep quality was not statistically significant (p=0.774) after the intervention. Figure 4 shows sleep hygiene according to the IHS-BR before and after the intervention.

*McNemar chi-squared test, significant if p-value < 0.05

Sleep hygiene showed a statistically significant improvement (p=0.012) after the intervention. Participants strengthened practices and habits which contributed to healthier sleep patterns, as they received guidance on how to sleep well. It is noted that there was an increase in the number of participants who demonstrated average/good sleep hygiene after the intervention.

The participants could select one or more options regarding descriptive data on self-reported symptoms before and after the intervention (n=40). There was a reduction in the percentages of symptoms after the intervention, namely: difficulty concentrating and/or memorizing, from 77.5% (n=31) to 22.5% (n=9); headache, from 57.5% (n=23) to 35% (n=14); decreased or increased appetite, from 50% (n=20) to 5% (n=2); irritability, from 50% (n=20) to 27.5% (n=11); feelings of indigestion or heartburn increased from 40% (n=16) to 7.5% (n=3); excessive daytime sleepiness increased from 40% (n=16) to 32.5% (n=13) and feelings of unhappiness increased from 25% (n=10) to 12.5% (n=5). 

Descriptive results regarding the degree of ease or difficulty participants observed in implementing each sleep hygiene habit indicated that keeping the bedroom dark, quiet and at a comfortable temperature for sleeping was considered easy to implement in the routine by 95% (n=38) of participants, while avoiding being in bed and performing tasks which stimulate brain activity was considered the most difficult to implement, affecting 37.5% (n=15) of participants. 

Based on data analysis, it was found that 100% (n=40) understood what sleep hygiene is and what the habits are; 87.5% (n=35) noticed improvements in their sleep hygiene routine/habits after the intervention and the same percentage identified that the intervention helped in some way to improve their sleep. Furthermore, 90% (n=36) indicated that reinforcement through text messages helped them adhere to the practice. Lastly, 75% (n=30) of the total reported that factors interfered with or hindered their sleep habits, such as family situations, work or specific academic issues during the study.

## Discussion

The educational intervention did not significantly alter sleep quality. Improving habits is essential for better sleep, but it does not always guarantee good sleep quality^10^. This can be explained by the fact that sleep is influenced by a combination of genetic, physiological, psychological, emotional, environmental and health factors^7^ ^,^ ^16^ ^-^ ^17^.

Furthermore, it is mentioned that excessive caffeine and alcohol consumption, inappropriate use of electronic devices, a sedentary lifestyle and lack of rest to the detriment of completing activities, deadlines, academic production and work and personal commitments can impair sleep and be associated with worry, anxiety and stress^1^ ^,^ ^4^ ^,^ ^18^. This can be seen in the results, as a greater number of students reported that certain factors interfered with or hindered sleep.

These factors can create a cycle in which sleep is frequently neglected, making it essential to pay attention to the sleep quality of nursing graduate students. In addition to harming physical and mental health, inadequate rest can also compromise these graduate students’ ability to perform their duties safely and effectively, especially when working in high-responsibility environments, such as healthcare institutions. This can lead to serious errors, as fatigue directly impacts attention and decision-making^3^. 

Furthermore, these individuals experienced a range of symptoms related to sleep deprivation. This indicates that inadequate rest can directly affect physical and emotional health^12^, which is concerning given the average age of 34, highlighting the need for sleep-related educational interventions to promote a future with healthier habits.

Nevertheless, the sleep hygiene of the graduate students showed a statistically significant improvement after the intervention. This suggests a connection with the participants’ increased knowledge about healthy habits, as found in another study^2^ which can be provided through the educational intervention.

The lack of knowledge about the physiological aspects of sleep and the consequences of sleep deprivation, given that these topics are not generally addressed during academic and professional training^19^, can foster bad habits, which are linked to health problems. Therefore, educational interventions become a relatively accessible and indispensable instruction form.

Beyond knowledge, the results suggest a practical implementation of these habits into the routines of graduate students. A significant increase was identified in the number of individuals who acquired sleep hygiene habits after the intervention, which is a feasible and satisfactory strategy for presenting and discussing the topic, enabling application of healthy habits in daily life based on the knowledge acquired. 

It is important to emphasize that implementing these habits considers each individual’s unique characteristics and is an ongoing process. Applying sleep hygiene habits consistently and practically is essential to ensure positive long-term effects. Improved sleep and health are directly linked to these habits, making it essential that they be considered a permanent commitment, not a temporary solution to sleep problems. This will help prevent harm and health problems, promoting quality of life and well-being^2^.

Corroborating the findings of this study, a study found that 90.3% of participants sleep in a dark and quiet environment, while 83.5% use their cell phones while lying in bed, constituting a habit that is unfavorable for sleep^20^. The discussion about the use of electronic devices before bed by graduate students deserves attention.

Research has shown that smartphone use in bed has significant adverse effects on sleep latency, wake time and heart rate in adults. This is due to blue light emission, which interferes with melatonin production and circadian rhythm through retinal ganglion cells (RGC) containing melanopsin (a receptor which measures the intensity of received light)^18^. When investigating the effect of reducing blue light from smartphone screens at night in young adults, research found that there was an improvement in subjective sleep quality, daytime functioning, and bedtime^21^.

One study evaluated the impact of blue light filtering on sleep quality, but found no sustained positive effects on these parameters^22^; another found no improvements in sleep and mental health in young adults after intervening with automated changes to the color temperature of electronic device screens^23^. Therefore, the impact of blue light filtering remains debatable. Even so, guidance on avoiding screen use before bed is essential.

Among some strategies to limit excessive exposure, it is essential to consciously distance yourself from digital devices. You can set time limits for smartphone, computer, laptop, tablet and television use, with functions, settings and apps available to help with this control. Set specific times for use, preferably during the day, refrain from staying connected at night and create designated “offline” times^24^. Moreover, avoid automatic use which occurs when there is no specific purpose for using the device and disable and/or manage notifications^24^. Furthermore, create pleasurable, screen-free spaces by seeking outdoor activities in the presence of family and friends, practicing hobbies, recreational activities, exercising and/or reading. Keep in mind that these strategies primarily depend on the interest, disposition, organization and limits of the individual seeking self-care.

Managing screen time before bed is part of preparing for rest, which includes adapting and adjusting the room where you sleep to promote rest. A study reinforces the idea of keeping the bedroom dark, quiet and at a mild temperature, which helps improve sleep duration^17^. It is a relatively easy and cost-effective practice that can be guided and yields positive results.

In this study, there was a significant improvement in sleep duration and subjective perception after the intervention. This demonstrates that objective measures and participants’ perceptions converge. This improvement is significant, given that adults generally need 7 to 9 hours of sleep per night. It is worth noting that there are specificities, as some adults feel comfortable with 6 hours of sleep, while others require 10 hours^9^.

It is important that each individual identify and respect their needs and limits, prioritizing maintaining the number of hours essential for adequate sleep. This result may be linked to a significant improvement in the perception of fatigue after the intervention. Adequate sleep resulting from a healthier routine positively impacts performance and mood.

Reinforcement through text messages and educational material provided with the content covered facilitated access to information and encouraged practice. Educational videos are widely used for health education and can bring health benefits and behavioral changes^25^. Similarly, a study which aimed to describe the development and validation of an educational booklet for nurses found that the material distributed online and in print represented a means of contributing to and strengthening practice. The layout of the content in texts, topics and illustrations encouraged use of the material, facilitating comprehension^26^.

The quasi-experimental study was chosen for its applicability, participant profile and community intervention. Biases in this study included a lack of randomization of participants, which may have led to differences in uncontrolled characteristics between groups, as well as a lack of control for external factors, such as personal, academic or work-related circumstances that could interfere with sleep. These factors were correlated with both the treatment and the outcome, potentially distorting the estimate of the causal effect.

Limitations include the type of study, which may suggest causal relationships, but the lack of randomization and control for external factors prevents definitive conclusions. Furthermore, the study was only conducted in one PPGEnf program in the State of Rio Grande do Sul and participants were lost during the study. The sample size and the difficulty in eliminating biases and confounding factors may limit generalizability of the results.

This study contributes to advance scientific knowledge in the health and nursing fields, as it presents an educational intervention on sleep hygiene as a comprehensive and low-cost practice which can be applied to a variety of contexts. Raising awareness among the population about the importance of healthy habits helps prevent and promote sleep, physical and mental health.

## Conclusion

The educational intervention improved sleep hygiene and subjective perception of sleep among students in the *stricto sensu* postgraduate program in Nursing. There was a significant increase in the number of students who acquired sleep hygiene habits after the intervention, with an increase in the number of hours of sleep and improvement in their perception of fatigue. There was no significant improvement in the sleep quality of the postgraduate students after the intervention.

## Data Availability

All data generated or analysed during this study are included in this published article.
