# Supplementary material for: Educational intervention on sleep hygiene habits and sleep quality of postgraduate students
Source: Rev Lat Am Enfermagem. 2026 Mar 16;34:e4765. doi: 10.1590/1518-8345.7996.4765 (PMC12991388; doi:10.1590/1518-8345.7996.4765)
Supplement: Translation

# Translation: Intervenção educativa sobre hábitos de higiene do sono e qualidade do sono de discentes pós-graduandos^2^

**DOI:** 10.1590/1518-8345.7996.4766

**Authors:** Ariane Naidon Cattani, Carmem Lúcia Colomé Beck, Rosângela Marion da Silva, Sandra Soares Mendes, Regina Célia Gollner Zeitoune, Silviamar Camponogara

**Affiliations:** 1 Universidade Federal de Santa Maria, Santa Maria, RS, Brasil.; 2 Bolsista do Conselho Nacional de Desenvolvimento Científico e Tecnológico (CNPq), Brasil.; 3 Centro Universitário das Faculdades Associadas de Ensino, Departamento de Enfermagem, São João da Boa Vista, SP, Brasil.; 4 Universidade Federal do Rio de Janeiro, Rio de Janeiro, RJ, Brasil

**Keywords:** Saúde do Trabalhador, Higiene do Sono, Qualidade do Sono, Sono, Programas de Pós-Graduação em Saúde, Enfermagem.

## Abstract

**Objetivo:**

analisar o efeito de uma intervenção educativa sobre os hábitos de higiene do sono e qualidade do sono de discentes da Pós-Graduação *stricto sensu* em Enfermagem.

**Método:**

estudo quase-experimental, do tipo antes e depois, com discentes de um Programa de Pós-Graduação em Enfermagem. Utilizou-se questionário sociolaboral, de hábitos de vida, de hábitos de sono, questionário para verificar a facilidade ou dificuldade para implementar as estratégias, o Índice de Qualidade do Sono de Pittsburgh e Índice de Higiene do Sono. A intervenção consistiu em sessão *online* com orientações sobre hábitos de higiene do sono, folheto e vídeo com o conteúdo apresentado e envio de mensagens de texto para reforçar as orientações. Para comparação antes e depois da intervenção, utilizou-se o teste McNemar com nível de significância de 5% (p<0,05).

**Resultados:**

participaram do estudo 40 pós-graduandos. Após a intervenção, a higiene do sono apresentou melhora (p=0,012). Os participantes aumentaram suas horas de sono (p=0,049), perceberam melhora no sono (p=0,016), adquiriram hábitos de higiene do sono (p<0,001) e houve redução no número de discentes que acordava se sentindo cansado (p=0,039).

**Conclusão:**

a intervenção melhorou a higiene do sono e a percepção subjetiva sobre o sono de pós-graduandos.

## Destaques

**(1)** A intervenção realizada melhorou a higiene do sono, a percepção subjetiva e o tempo total do sono. **(2)** A intervenção resultou em melhorias nos hábitos de sono e percepção sobre o cansaço. **(3)** Higiene do sono é uma prática acessível, que possibilita o autocuidado.

## Introdução

O sono é uma necessidade humana básica que, quando adequado, promove bem-estar, saúde física e mental às pessoas. Apesar de ser imprescindível para os seres humanos, estima-se que 20 a 45% da população mundial possui privação de sono^1^, o que representa um desafio crescente para a Saúde Pública.

Fatores biológicos e ambientais, associados aos comportamentos e hábitos inadequados, podem acarretar tempo de descanso insuficiente e sonolência diurna excessiva, além de prejuízos na vida diária das pessoas, uma vez que interferem no rendimento e na qualidade de vida^2^. Essas situações podem gerar alto impacto social e econômico, utilização de recursos médicos e serviços farmacêuticos, além das despesas relacionadas ao absenteísmo, redução de produção acadêmica, no trabalho e aumento de acidentes laborais^3^.

Discentes de pós-graduação em enfermagem, como em outras pós-graduações, possuem alta demanda acadêmica, laboral e social, além de enfrentarem dificuldades relacionadas ao sofrimento mental, falta de apoio financeiro à pesquisa e acúmulo de vínculos empregatícios com as atividades do curso^4^. Destaca-se que esses são, majoritariamente, enfermeiros, que estão expostos a um ambiente laboral complexo e precisam lidar com o fato de cuidar da saúde e vida de outras pessoas. Esse aspecto exige atenção, concentração, tomada de decisão rápida e necessidade de estudo e atualizações contínuas, o que demanda tempo e diminui as horas para repouso^3^.

O contexto pode ser agravado por maus hábitos diários, como a ingestão excessiva e inadequada de substâncias estimulantes, o uso demasiado de telas como *smartphones*, computadores e *tablets*, estudar, trabalhar e realizar atividades que demandam alta atividade cerebral na cama ou antes de dormir. A privação de sono e a dificuldade para manter uma rotina adequada prejudicam o desempenho cognitivo, memória, concentração, o sistema cardiovascular, metabólico, imunológico e hormonal, favorecendo sintomas de ansiedade e depressão com prejuízos à saúde emocional^1^. 

Diante desses fatos, ressalta-se a importância de realizar intervenções que objetivem melhorias relacionadas ao sono e à saúde, por meio do autocuidado. Sugere-se a prática de higiene do sono, utilizada em outros estudos que evidenciaram melhora no sono em geral^2^ ^,^ ^5^ ^-^ ^6^. 

A prática refere-se à adoção de hábitos básicos que podem auxiliar um padrão de sono mais saudável. Pode ser orientada por meio de uma intervenção educativa, que objetiva explicar técnicas comportamentais e de controle de estímulos para reduzir fatores prejudiciais ao sono, que incluem latência, duração, qualidade e percepção subjetiva do sono^7^. Sugere-se que as orientações sejam realizadas de forma verbal e escrita^8^.

Os hábitos incluem manter um horário de dormir e acordar regular, evitar o consumo de substâncias estimulantes antes de dormir, praticar exercícios físicos regularmente e manter o ambiente agradável para o repouso^8^ ^-^ ^9^. Uma intervenção educativa sobre higiene do sono é uma das formas de educar pessoas para o autocuidado^10^. 

Para aprofundar o conhecimento sobre intervenções realizadas para melhorar o sono, realizou-se uma revisão integrativa no ano de 2024, produto de disciplina vinculada à pós-graduação. Não se identificou estudo com discentes pós-graduandos, apontando uma lacuna na produção do conhecimento, o que ressalta a necessidade de estudos de intervenção voltados para essa população.

Dito isso, buscou-se elucidar a pergunta de pesquisa: realizar uma intervenção educativa sobre higiene do sono, melhora os hábitos e a qualidade do sono de discentes da Pós-graduação *stricto sensu* em Enfermagem? Tem-se como objetivo analisar o efeito de uma intervenção educativa sobre os hábitos de higiene do sono e qualidade do sono de discentes da Pós-Graduação *stricto sensu* em Enfermagem.

## Método

### Tipo do estudo

Trata-se de um estudo quase-experimental do tipo antes e depois, não randomizado, no qual foi realizada uma intervenção educativa *online* sobre hábitos de higiene do sono. O estudo seguiu as orientações da lista de verificação do *Strengthening the Reporting of Observational Studies in Epidemiology (Standards for Quality Improvement Reporting Excellence)* (SQUIRE 2.0).

### Local do estudo

Foi realizado em um Programa de Pós-Graduação *stricto sensu* em Enfermagem (PPGEnf) de uma Universidade Federal localizada no Rio Grande do Sul, Brasil. O curso de mestrado possuía carga horária total de 360h, enquanto o de doutorado totalizava 600 h. Os discentes realizavam as disciplinas obrigatórias e optativas, elaboravam e defendiam sua dissertação ou tese, participavam de atividades dos grupos de pesquisa, do programa e de eventos científicos. O ingresso ocorria anualmente, por orientador e, em média, eram ofertadas 22 vagas para cada curso. 

### População e amostra do estudo

Foi realizado com discentes dos cursos de mestrado e doutorado do Programa de Pós-Graduação *stricto sensu* em Enfermagem (PPGEnf). No período da coleta de dados, estavam matriculados 133 discentes, sendo 64 mestrandos e 69 doutorandos. Optou-se pela amostragem por conveniência (não probabilística) devido à disponibilidade dos participantes. A coleta de dados ocorreu de maio de 2023 a fevereiro de 2024.

### Critérios de seleção

Estipulou-se como critérios de inclusão: possuir entre 18 e 59 anos de idade, devido às particularidades relacionadas ao sono em cada faixa etária e estar matriculado regularmente no PPGEnf; como critério de exclusão: estar em licença ou afastamento de qualquer natureza durante o período da coleta de dados. É oportuno esclarecer que os participantes não foram questionados previamente à entrega dos instrumentos de coleta de dados se utilizavam medicação com efeitos sobre o sono, apneia/hipopneia, distúrbios neuropsiquiátricos ou dor crônica. 

### Intervenção educativa

A intervenção educativa consistiu em uma sessão *online* em que uma das autoras, pós-graduanda com experiência no tema e em coleta de dados quantitativos, forneceu orientações verbais, por meio de exposição dialogada, com auxílio de uma apresentação sobre aspectos gerais, fases e importância do sono, ritmo circadiano e possíveis prejuízos à saúde física, mental e emocional decorrentes de alterações no sono. Também, sobre hábitos de higiene do sono, apresentando o conceito, comportamentos pessoais e fatores ambientais que podem auxiliar a sua melhoria. Os participantes foram convidados a implementar esses hábitos na sua rotina diária.

O conteúdo abordado na intervenção educativa foi embasado em evidências científicas identificadas na revisão integrativa previamente realizada e nas diretrizes da Associação Brasileira do Sono^8^ ^-^ ^9^, adaptadas para a população deste estudo. Incluíram orientações como ir para a cama, à noite, quando estiver sonolento; manter uma rotina regular no horário de deitar e levantar (independentemente do horário que foi dormir), inclusive no fim de semana; evitar permanecer mais do que 30 minutos na cama se acordado; se precisar levantar, evitar luz branca ou azul (encontrar um lugar para relaxar fora do dormitório e só retornar para cama com sono, mesmo durante a madrugada).

Evitar utilizar a cama para tarefas que estimulem a atividade cerebral (ler, trabalhar, assistir TV, utilizar o celular ou qualquer tela que emita luminosidade); evitar atividades estressantes ao deitar-se, podendo anotar os compromissos na agenda e reservar um período durante o dia para se concentrar nos problemas que preocupam; evitar cochilar durante o dia (caso seja necessário, limitar a duração a aproximadamente 30 minutos, após o almoço).

Restringir o uso de álcool, nicotina e substâncias estimulantes (café, chimarrão, energéticos) após as 15 h; evitar comer em excesso dentro de, aproximadamente, quatro horas antes de se deitar; praticar exercícios físicos regularmente (até uma hora antes do horário de dormir); tomar banho morno próximo a hora de se deitar para relaxar; praticar exercícios de controle da respiração a qualquer momento do dia para relaxar; manter o ambiente onde dorme escuro, silencioso e com temperatura agradável.

As sessões *online* ocorreram nos diferentes turnos e foram realizadas um total de 21 sessões via *Google Meet*. Cada participante foi alocado em um grupo para participar da sessão, conforme escolha da data e horário previamente informados, optou-se pela formação de grupos para permitir discussões. No turno da manhã, foram cinco grupos, no turno da tarde 12 grupos e no turno da noite quatro grupos. Os grupos obtiveram no mínimo 1 e no máximo 7 participantes e cada indivíduo participou uma vez. Apesar do quantitativo variável de participantes em cada encontro, a intervenção seguiu rigorosamente um roteiro previamente elaborado. 

Cada encontro teve duração aproximada de 25-30 minutos. Ao final de cada encontro, foi disponibilizado, aproximadamente, 10 minutos para os participantes compartilharem suas experiências, dificuldades e facilidades relacionadas às suas rotinas e sono, bem como esclarecerem dúvidas a respeito do tema.

Imediatamente após finalizar cada sessão *online*, foi enviado via *Whatsapp* ^*®*^ , para cada participante que estava presente no encontro, um folheto em *Portable Document Format* (PDF) e um vídeo de 1 minuto e 20 segundos, ambos elaborados pelos autores, com as orientações sobre higiene do sono, possibilitando o acesso ao conteúdo sempre que desejado.

Foram enviadas quatro mensagens de texto, uma por semana, incentivando e relembrando sobre os hábitos, oportunizando esclarecimento de possíveis dúvidas e compartilhamento da experiência. A primeira mensagem foi enviada sete dias após a participação na intervenção educativa; a segunda mensagem, sete dias após a primeira e assim consecutivamente.

### Instrumentos

Utilizou-se um questionário sociolaboral com variável quantitativa relacionada à idade, e variáveis qualitativas, categorizadas em sim ou não, relacionadas ao sexo atribuído no nascimento, filhos, pós-graduação, deslocamento para a pós-graduação e trabalho; questionário de hábitos de vida, com variáveis qualitativas, também categorizadas em sim ou não, sobre o uso de medicações para dormir e sintomas autorrelatados de saúde; questionário sobre hábitos de sono, com variáveis sobre duração, latência, percepção sobre o sono, sonolência, hábitos de higiene do sono e rotina de dormir.

Para verificar o grau de facilidade ou dificuldade para adormecer, elaboraram-se questões onde o participante assinalava a opção “fácil” ou “difícil” para perguntas como: “Como foi, para você, à noite, ir para a cama quando estava sonolento? Como foi, para você, manter uma rotina regular no horário de deitar e levantar?”. Essas questões foram elaboradas por uma pós-graduanda do curso de doutorado e analisadas por duas professoras doutoras, com experiência em pesquisas sobre a saúde do trabalhador e sono. 

Para avaliar a qualidade do sono, foi utilizado o Índice de Qualidade do Sono de Pittsburgh, versão validada em português do Brasil (PSQI-BR), com escore global que pode variar de 0 a 21 pontos, classificando como qualidade do sono boa (≤ 4), qualidade do sono ruim (5-10) e presença de distúrbio do sono (≥ 11)^11^. Optou-se por categorizar a variável em qualidade do sono “boa” (≤ 5) e “ruim” (> 5 pontos), conforme utilizado em outro estudo^12^.

Para mensurar os hábitos de higiene do sono, foi utilizado o Índice de Higiene do Sono, versão validada em português do Brasil (IHS-BR). Sua pontuação pode variar de 13 a 65 pontos, sendo as pontuações mais altas um indicativo de higiene do sono inadequada^13^. Para este estudo, foi considerada higiene do sono “média/boa” (≤ 34 pontos) e “ruim” (≥35 pontos)^14^ ^-^ ^15^.

Houve teste-piloto com os instrumentos de coleta de dados junto aos participantes de um grupo de pesquisa para verificar a clareza das perguntas, tempo de preenchimento e possíveis dificuldades de interpretação. Após ajustes na formatação, os instrumentos foram considerados adequados para a coleta. 

### Coleta de dados

A coleta de dados ocorreu de forma *online,* no *Google Forms*, em três etapas: antes, na implementação e após a intervenção. A intervenção era ofertada após o preenchimento dos questionários da pré-intervenção e consistia na exposição dialogada e no envio de quatro mensagens de texto como reforço dos hábitos de higiene do sono. Os questionários da pós-intervenção eram disponibilizados uma semana após o envio da quarta e última mensagem.

Para melhor visualização das etapas do desenvolvimento do estudo, apresenta-se um fluxograma na Figura 1.

**Figura 1:** Fluxograma do desenvolvimento da pesquisa

### Análise dos dados

Os dados coletados foram exportados do *Google Forms*, organizados no *software Microsoft Excel* ^*®*^ e analisados no programa *Statistical Package for the Social Sciences* ^*®*^ (SPSS 25.0). As variáveis categóricas estão descritas pela frequência absoluta (N) e relativa (%) e as quantitativas pela média e desvio-padrão. A alteração entre os momentos, para cada hábito do sono, qualidade sono e índice de higiene do sono, foi descrita e comparada antes e depois da intervenção com o uso do teste Qui-quadrado de McNemar, considerando-se para a rejeição da hipótese de nulidade um nível de significância de 5% (p<0,05).

### Aspectos éticos

Os aspectos éticos foram respeitados, conforme recomendações da Resoluções 466/12 e 510/2016 do Conselho Nacional de Saúde. O estudo foi aprovado pelo Comitê de Ética em Pesquisa da Universidade Federal de Santa Maria, com Certificado de Apresentação de Apreciação Ética nº 66359622.6.0000.5346, sob o Parecer nº 5.847.306 de janeiro de 2023. Menciona-se que no Termo de Consentimento Livre e Esclarecido constava a informação sobre o uso de mensagens de texto via *WhatsApp* ^*®*^ .

## Resultados

Responderam os questionários da pré intervenção, 53 pós-graduandos em enfermagem (39,84% da população); dentre esses, 11 não participaram da intervenção e dois não responderam os questionários da pós-intervenção. Completaram todas as fases do estudo, 40 participantes, considerados para a análise comparativa, que possuíam média de idade de 34 anos (desvio-padrão±8,11) (mínimo, 23; máximo, 54 anos), 95% (n=38) atribuíram-se o feminino e 42,5% (n=17) possuíam filhos. Dos participantes, 50% (n=20) realizavam doutorado, sendo que 25% (n=10) precisavam se deslocar para outra cidade ou estado para realizar a pós-graduação, 87,5% (n=35) trabalhavam, 37,5% (n=15) realizavam algum tratamento de saúde e 20% (n=8) utilizavam medicação para dormir.

Foram realizadas análises antes e depois da intervenção (n=40), nas quais se comparou os hábitos de sono, a qualidade de sono (PSQI-BR) e a higiene do sono (IHS-BR) dos participantes.

A seguir, a Tabela 1 representa a comparação antes e depois da intervenção entre variáveis relacionadas aos hábitos de sono dos discentes de pós-graduação.

**Tabela 1:** Variáveis relacionadas aos hábitos de sono antes e depois da intervenção com discentes pós-graduandos (n = 40). Santa Maria, RS, Brasil, 2023-2024

Variável	Antes da intervenção n (%)	Depois da intervenção n (%)	p-valor*
Considera suficientes as horas de sono			
Sim	16 (40,0)	24 (60,0)	0,057
Não	24 (60,0)	16 (40,0)	
Dificuldade em manter o sono			
Sim	15 (37,5)	9 (22,5)	0,146
Não	25 (62,5)	31 (77,5)	
Dificuldade em iniciar o sono			
Sim	13 (32,5)	8 (20,0)	0,125
Não	27 (67,5)	32 (80,0)	
Dificuldade em acordar			
Sim	23 (57,5)	16 (40,0)	0,065
Não	17 (42,5)	24 (60,0)	
Sonolência durante o dia			
Sim	26 (65,0)	22 (55,0)	0,388
Não	14 (35,0)	18 (45,0)	
Total	40 (100)	40 (100)	

*Teste Qui-quadrado de McNemar, significativo se p-valor < 0,05

Não foi identificada significância estatística nesses hábitos de sono. A seguir, na Figura 2, pode-se visualizar diferença estatística entre a quantidade de horas de sono, a percepção sobre o sono, a sensação de cansaço e possuir hábitos de higiene do sono antes e depois da intervenção.

**Figura 2:** Hábitos de sono antes e depois da intervenção com discentes pós-graduandos (n = 40). Santa Maria, RS, Brasil, 2023-2024

*Teste Qui-quadrado de McNemar, significativo se p-valor < 0,05

Após a intervenção, houve aumento percentual de participantes que elevaram suas horas de sono de < 7h para ≥ 7h a 9h por noite (p=0,049) (22,5%), que perceberam melhora sobre seu sono (p=0,016) (17,5%) e que adquiriram hábitos de higiene do sono (p<0,001) (67,5%). Observou-se redução percentual de discentes que acordavam sentindo-se cansados (p=0,039) (20,2%). 

A Figura 3 mostra a qualidade do sono, conforme o PSQI-BR, antes e após a intervenção.

**Figura 3:** Qualidade do sono antes e depois da intervenção com discentes pós-graduandos (n = 40). Santa Maria, RS, Brasil, 2023-2024

*Teste Qui-quadrado de McNemar, significativo se p-valor < 0,05

A qualidade do sono não apresentou significância estatística (p=0,774) após a intervenção. Na sequência, a Figura 4 mostra a higiene do sono, de acordo com o IHS-BR, antes e após a intervenção.

**Figura 4:** Higiene do sono antes e depois da intervenção com discentes pós-graduandos (n = 40). Santa Maria, RS, Brasil, 2023-2024

*Teste Qui-quadrado de McNemar, significativo se p-valor < 0,05

A higiene do sono apresentou melhora estatisticamente significativa (p=0,012) após a intervenção, ou seja, os participantes fortaleceram as práticas e hábitos que auxiliaram no padrão de sono mais saudável, pois receberam orientação para dormir bem. Menciona-se que houve aumento no número de participantes que apresentaram média/boa higiene do sono após a intervenção.

Em relação aos dados descritivos sobre os sintomas autorrelatados antes e também após a intervenção (n=40), os participantes puderam marcar uma ou mais opções. Após a intervenção, houve redução nos percentuais dos sintomas: dificuldade de concentração e/ou memorização, de 77,5% (n=31) para 22,5% (n=9); dor de cabeça, de 57,5% (n=23) para 35% (n=14); diminuição ou aumento de apetite, de 50% (n=20) para 5% (n=2); irritabilidade, de 50% (n=20) para 27,5% (n=11); sensação de má digestão ou azia, de 40% (n=16) para 7,5% (n=3); sonolência diurna excessiva, de 40% (n=16) para 32,5% (n=13) e sensação de infelicidade, de 25% (n=10) para 12,5% (n=5).

Os resultados descritivos a respeito do grau de facilidade ou dificuldade que os participantes observaram na implementação de cada hábito de higiene do sono apontaram que manter o quarto escuro, silencioso e com temperatura agradável para dormir, foi observado como de fácil implementação na rotina por 95% (n=38) dos participantes, enquanto evitar a cama para realizar tarefas que estimulem a atividade cerebral foi considerado o mais difícil de implementar-se, contemplando 37,5% (n=15) dos participantes. 

A partir da análise dos dados, identificou-se que 100% (n=40) compreenderam o que é higiene do sono e quais são os hábitos; 87,5% (n=35) perceberam melhoria na rotina/hábitos de higiene do sono após intervenção e a mesma porcentagem identificou que a intervenção auxiliou de alguma forma na melhoria do sono. Além disso, 90% (n=36) apontaram que o reforço, por meio das mensagens de texto, auxiliou na adesão à prática. 

Do total, 75% (n=30) relataram que houve fatores que interferiram ou dificultaram seus hábitos de sono, como situações familiares, questões laborais ou acadêmicas específicas durante o desenvolvimento do estudo.

## Discussão

A intervenção educativa não alterou significativamente a qualidade do sono. Melhorar hábitos é um processo essencial para dormir melhor, mas nem sempre garante uma boa qualidade do sono^10^. Isso pode ser explicado pelo fato de que o sono é influenciado por uma combinação de fatores genéticos, fisiológicos, psicológicos, emocionais, ambientais e condições de saúde^7^ ^,^ ^16^ ^-^ ^17^. 

Ainda, menciona-se que o consumo excessivo de cafeína, álcool, utilização inadequada de dispositivos eletrônicos, sedentarismo, a própria privação do descanso em detrimento ao cumprimento de atividades, prazos, produção acadêmica, compromissos laborais e pessoais podem prejudicar o sono e estar associados com preocupação, ansiedade e estresse^1^ ^,^ ^4^ ^,^ ^18^. Isso pode ser observado nos resultados, pois um maior quantitativo de discentes relatou que houve fatores que interferiram ou dificultaram o sono.

Esses fatores podem criar um ciclo no qual o sono é frequentemente negligenciado, o que torna imprescindível a atenção à qualidade do sono dos pós-graduandos em enfermagem. O descanso inadequado, além de prejudicar a saúde física e mental, pode comprometer a capacidade desses pós-graduandos em desempenharem suas funções de forma segura e eficaz, especialmente quando atuam em ambientes de alta responsabilidade, como instituições de saúde. Isso pode levar aos erros graves, uma vez que a fadiga impacta diretamente na atenção e na tomada de decisões^3^. 

Além disso, esses indivíduos enfrentavam uma série de sintomas relacionados à privação de sono. Isso indica que o repouso inadequado pode afetar diretamente a saúde física e emocional^12^, o que preocupa ao se levar em conta a média de idade de 34 anos, indicando a necessidade de intervenções educativas sobre o sono para promover uma perspectiva de futuro com hábitos mais saudáveis.

Contudo, após a intervenção, a higiene do sono dos pós-graduandos apresentou melhora estatisticamente significativa. Isso sugere relação com o aumento do conhecimento dos participantes sobre hábitos saudáveis, como encontrado em outra pesquisa^2^, o que pode ser ofertado por meio da intervenção educativa realizada. 

A falta de conhecimento sobre aspectos fisiológicos do sono e as consequências de sua privação, visto que não são temas abordados durante a formação acadêmica e profissional em geral^19^, pode favorecer maus hábitos, o que está atrelado aos danos à saúde. Assim, intervenções educativas tornam-se uma forma de instrução relativamente acessível e indispensável.

Para além do conhecimento, a partir dos resultados sugere-se a implementação de forma prática dos hábitos na rotina dos pós-graduandos. Identificou-se aumento expressivo no número de indivíduos que adquiriram hábitos de higiene do sono após a intervenção, sendo uma estratégia factível e satisfatória para apresentar e discutir o tema, viabilizando a aplicação de hábitos saudáveis no cotidiano a partir do conhecimento adquirido. 

Destaca-se que, a implementação dos hábitos considera as particularidades de cada indivíduo, sendo um processo contínuo. Aplicar os hábitos de higiene do sono, de forma prática e constante, é fundamental para garantir efeitos positivos ao longo prazo. A melhora do sono e da saúde está diretamente ligada a esses hábitos, o que torna essencial que eles sejam considerados um compromisso permanente e não uma solução temporária para problemas de sono. Assim, auxiliará na prevenção de danos e de agravos à saúde, promovendo qualidade de vida e bem-estar^2^. 

Corroborando os achados do presente estudo, uma pesquisa identificou que 90,3% dos participantes dormiam em ambiente escuro e silencioso, enquanto 83,5% utilizavam o celular deitados na cama, sendo um hábito desfavorável para o sono^20^. A discussão acerca do uso de dispositivos eletrônicos antes de dormir por discentes pós-graduandos merece atenção.

Pesquisa apontou que o uso do *smartphone* na cama tem efeitos adversos significativos na latência do sono, no tempo de vigília e na frequência cardíaca de adultos. Isso devido a emissão de luz azul, que interfere na produção de melatonina e no ritmo circadiano por meio de células ganglionares da retina (RGC) contendo melanopsina (receptor que mede a intensidade da luz recebida)^18^. Ao investigar o efeito da redução da luz azul da tela do *smartphone*, durante a noite, em adultos jovens, pesquisa identificou que houve melhoria na qualidade subjetiva do sono, no funcionamento diurno e na hora de dormir^21^.

Estudo avaliou o impacto da aplicação do filtro de luz azul na qualidade do sono, porém não encontrou efeitos positivos sustentados nesses parâmetros^22^; outro não encontrou melhorias no sono e na saúde mental em adultos jovens após intervir com alterações automatizadas na temperatura de cor da tela de dispositivos eletrônicos^23^, ou seja, o impacto de filtragem de luz azul permanece discutível. Assim, torna-se imprescindível a orientação sobre evitar o uso de telas antes de dormir. 

Dentre algumas estratégias para restringir a exposição excessiva, é primordial ter o hábito de afastar-se conscientemente dos aparelhos digitais. Pode-se estabelecer limites de tempo para o uso de *smartphone*, computador, *notebook, tablet* e televisão, havendo funções, configurações e aplicativos disponíveis para auxiliar nesse controle. Determinar horários específicos para o uso, preferencialmente durante o dia, abstendo-se de manter-se conectado à noite e criando horários designados “*offline*”^24^.

Evitar o uso automático, que ocorre quando não se tem um objetivo específico para utilizar o dispositivo; aliado a isso, desativar e/ou gerenciar notificações^24^. Também, produzir espaços prazerosos e sem telas, buscando realizar atividades ao ar livre, na presença de familiares e amigos, praticando hobbies, realizando atividades recreativas, exercícios físicos e/ou leituras. Lembrando que essas estratégias dependem principalmente do interesse, disposição, organização e limites do indivíduo que busca o autocuidado. 

O gerenciamento das telas antes de dormir entra no contexto de preparar-se para o momento de repouso, o que inclui adequar e adaptar o cômodo onde se dorme, favorecendo o descanso. Estudo reforça o fato de manter o quarto escuro, silencioso e a temperatura amena, auxiliando na duração do sono^17^. É uma prática relativamente fácil e sem custo, que pode ser orientada, apresentando resultados positivos.

Após a intervenção, houve melhora significativa nas horas e percepção subjetiva sobre o sono. Isso retrata que as medidas objetivas e a percepção dos participantes convergem. Essa melhora é relevante visto que, em geral, é necessário de 7 a 9h de sono por noite para adultos. Vale ressaltar que há particularidades, pois alguns adultos sentem-se bem com 6h de sono, enquanto outros necessitam de 10h^9^.

É importante que cada indivíduo identifique e respeite suas necessidades e limites, priorizando manter a quantidade de horas indispensáveis para um sono adequado. Esse resultado pode estar atrelado à melhora significativa na percepção sobre o cansaço após a intervenção. A quantidade de sono adequada, resultante de uma rotina mais saudável, interfere positivamente no desempenho e disposição.

O reforço, por meio de mensagens de texto e o material educativo disponibilizado com o conteúdo abordado facilitou o acesso às informações e estimulou a prática. Vídeos educativos são amplamente utilizados para a educação em saúde da população, podendo trazer benefícios para a saúde e mudanças comportamentais^25^.

Da mesma forma, um estudo que objetivou descrever a elaboração e validação de uma cartilha educativa para enfermeiros, identificou que o material distribuído de forma *online* e impressa, representou um meio de contribuição e fortalecimento da prática. A diagramação do conteúdo em textos, tópicos, ilustrações impulsionaram a utilização do material, facilitando a compreensão^26^.

A opção pelo estudo quase-experimental deu-se pela aplicabilidade, perfil dos participantes e por ser uma intervenção comunitária. Os vieses desse estudo envolveram a não randomização dos participantes, em que os grupos podem diferir em características que não foram controladas; houve a ausência de controle de fatores externos, como a ocorrência de situações pessoais, acadêmicas ou laborais que poderiam interferir no sono, correlacionados tanto com o tratamento quanto com o desfecho, podendo distorcer a estimativa do efeito causal. 

As limitações referem-se ao tipo de estudo que pode sugerir relações causais, mas a falta de randomização e de controle de fatores externos impede uma conclusão definitiva. Também, ao fato de o estudo ter sido realizado em apenas um PPGEnf do estado do Rio Grande do Sul e ainda, a perda de participantes durante o desenvolvimento da pesquisa. O tamanho da amostra e a dificuldade de eliminar vieses e fatores confundidores pode limitar a generalização dos resultados.

Esse estudo contribui para o avanço do conhecimento científico na área da saúde e enfermagem, pois apresenta uma intervenção educativa sobre higiene do sono como uma prática abrangente e de baixo custo, que se aplica aos diversos contextos. Sensibilizar a população sobre a importância de hábitos saudáveis colabora com a prevenção e promoção do sono, saúde física e mental.

## Conclusão

A intervenção educativa melhorou a higiene do sono e a percepção subjetiva sobre o sono de discentes da Pós-graduação *stricto sensu* em Enfermagem. Após a intervenção, houve aumento significativo no quantitativo de discentes que adquiriram os hábitos de higiene do sono, com aumento na quantidade de horas de sono e melhora na percepção sobre o cansaço. Não houve melhora significativa na qualidade do sono dos pós-graduandos, após a intervenção. 

## Declaração de Disponibilidade de Dados:

Todos os dados gerados ou analisados durante este estudo estão incluídos neste artigo publicado.

# Translation: Intervención educativa sobre hábitos de higiene del sueño y calidad del sueño de estudiantes de posgrado^3^

**DOI:** 10.1590/1518-8345.7996.4764

**Authors:** Ariane Naidon Cattani, Carmem Lúcia Colomé Beck, Rosângela Marion da Silva, Sandra Soares Mendes, Regina Célia Gollner Zeitoune, Silviamar Camponogara

**Affiliations:** 1 Universidade Federal de Santa Maria, Santa Maria, RS, Brasil.; 2 Becaria del Conselho Nacional de Desenvolvimento Científico e Tecnológico (CNPq), Brasil.; 3 Centro Universitário das Faculdades Associadas de Ensino, Departamento de Enfermagem, São João da Boa Vista, SP, Brasil.; 4 Universidade Federal do Rio de Janeiro, Rio de Janeiro, RJ, Brasil.

**Keywords:** Salud del Trabajador, Higiene del Sueño, Calidad del Sueño, Sueño, Programas de Posgrado en Salud, Enfermería.

## Abstract

**Objetivo:**

analizar el efecto de una intervención educativa sobre los hábitos de higiene del sueño y la calidad del sueño de estudiantes de posgrado *stricto sensu* en Enfermería

**Método:**

estudio cuasi experimental, de tipo antes y después, con estudiantes de un Programa de Posgrado en Enfermería. Se utilizaron un cuestionario sociolaboral, de hábitos de vida, de hábitos de sueño, un cuestionario para verificar la facilidad o dificultad para implementar las estrategias, el Índice de Calidad del Sueño de Pittsburgh y el Índice de Higiene del Sueño. La intervención consistió en una sesión en línea con orientaciones sobre hábitos de higiene del sueño, folleto y video con el contenido presentado y envío de mensajes de texto para reforzar las orientaciones. Para la comparación antes y después de la intervención, se empleó la prueba de McNemar con un nivel de significancia del 5% (p<0,05).

**Resultados:**

participaron en el estudio 40 estudiantes de posgrado. Tras la intervención, la higiene del sueño mostró mejora (p=0,012). Los participantes aumentaron sus horas de sueño (p=0,049), percibieron mejora en el sueño (p=0,016), adquirieron hábitos de higiene del sueño (p<0,001) y hubo reducción en el número de estudiantes que se despertaban sintiéndose cansados (p=0,039).

**Conclusión:**

la intervención mejoró la higiene del sueño y la percepción subjetiva sobre el sueño de estudiantes de posgrado.

## Destacados

(1) La intervención realizada mejoró la higiene del sueño, la percepción subjetiva y el tiempo total de sueño. (2) La intervención resultó en mejoras en los hábitos de sueño y en la percepción sobre el cansancio. (3) La higiene del sueño es una práctica accesible que posibilita el autocuidado.

## Introducción

El sueño es una necesidad humana básica que, cuando es adecuado, promueve el bienestar y la salud física y mental de las personas. A pesar de ser imprescindible para los seres humanos, se estima que 20 a 45% de la población mundial presenta privación de sueño^1^, lo que representa un desafío creciente para la Salud Pública.

Factores biológicos y ambientales, asociados a comportamientos y hábitos inadecuados, pueden acarrear tiempo de descanso insuficiente y somnolencia diurna excesiva, además de perjuicios en la vida diaria de las personas, dado que interfieren en el rendimiento y en la calidad de vida^2^. Estas situaciones pueden generar alto impacto social y económico, utilización de recursos médicos y servicios farmacéuticos, además de los gastos relacionados con el absentismo, la reducción de la producción académica y en el trabajo y el aumento de accidentes laborales^3^.

Los estudiantes de posgrado en Enfermería, como en otros posgrados, poseen alta demanda académica, laboral y social, además de enfrentar dificultades relacionadas con el sufrimiento mental, la falta de apoyo financiero a la investigación y la acumulación de vínculos laborales con las actividades del curso^4^. Se destaca que estos son, mayoritariamente, enfermeros, que están expuestos a un entorno laboral complejo y deben lidiar con el hecho de cuidar la salud y la vida de otras personas. Este aspecto exige atención, concentración, toma de decisiones rápida y necesidad de estudio y actualizaciones continuas, lo que demanda tiempo y disminuye las horas para el descanso^3^.

El contexto puede verse agravado por malos hábitos diarios, como la ingestión excesiva e inadecuada de sustancias estimulantes, el uso excesivo de pantallas como *smartphones*, computadoras y tabletas, estudiar, trabajar y realizar actividades que demandan alta actividad cerebral en la cama o antes de dormir. La privación de sueño y la dificultad para mantener una rutina adecuada perjudican el desempeño cognitivo, la memoria, la concentración y los sistemas cardiovascular, metabólico, inmunológico y hormonal, favoreciendo síntomas de ansiedad y depresión con perjuicios para la salud emocional^1^.

Ante estos hechos, se resalta la importancia de realizar intervenciones que tengan por objetivo mejoras relacionadas con el sueño y la salud, por medio del autocuidado. Se sugiere la práctica de higiene del sueño, utilizada en otros estudios que evidenciaron mejora en el sueño en general^2^ ^,^ ^5^ ^-^ ^6^. La práctica se refiere a la adopción de hábitos básicos que pueden auxiliar un patrón de sueño más saludable. Puede orientarse por medio de una intervención educativa, que tiene por objetivo explicar técnicas conductuales y de control de estímulos para reducir factores perjudiciales para el sueño, que incluyen latencia, duración, calidad y percepción subjetiva del sueño^7^. Se sugiere que las orientaciones se realicen de forma verbal y escrita^8^.

Los hábitos incluyen mantener un horario regular para dormir y despertar, evitar el consumo de sustancias estimulantes antes de dormir, practicar ejercicios físicos regularmente y mantener un entorno agradable para el descanso^8^ ^-^ ^9^. Una intervención educativa sobre higiene del sueño es una de las formas de educar a las personas para el autocuidado^10^.

Para profundizar el conocimiento sobre intervenciones realizadas para mejorar el sueño, se llevó a cabo una revisión integrativa en el año 2024, producto de una asignatura vinculada al posgrado. No se identificó estudio con estudiantes de posgrado, lo que señala una laguna en la producción de conocimiento y resalta la necesidad de estudios de intervención dirigidos a esa población.

Dicho esto, se buscó dilucidar la pregunta de investigación: realizar una intervención educativa sobre higiene del sueño, ¿mejora los hábitos y la calidad del sueño de estudiantes del posgrado *stricto sensu* en Enfermería? Se tiene como objetivo analizar el efecto de una intervención educativa sobre los hábitos de higiene del sueño y la calidad del sueño de estudiantes del posgrado *stricto sensu* en Enfermería.

## Método

### Tipo del estudio

Se trata de un estudio cuasi experimental de tipo antes y después, no aleatorizado, en el cual se realizó una intervención educativa en línea sobre hábitos de higiene del sueño. El estudio siguió las orientaciones de la lista de verificación del *Strengthening the Reporting of Observational Studies in Epidemiology* (*Standards for Quality Improvement Reporting Excellence*) (SQUIRE 2.0).

### Lugar del estudio

Se realizó en un Programa de Posgrado *stricto sensu* en Enfermería (PPGEnf) de una Universidad Federal ubicada en Rio Grande do Sul, Brasil. El curso de maestría tenía una carga horaria total de 360h, mientras que el de doctorado totalizaba 600h. Los estudiantes cursaban asignaturas obligatorias y optativas, elaboraban y defendían su disertación o tesis, participaban en actividades de los grupos de investigación, del programa y de eventos científicos. El ingreso ocurría anualmente, por orientador y, en promedio, se ofrecían 22 plazas para cada curso.

### Población y muestra del estudio

Se realizó con estudiantes de los cursos de maestría y doctorado del Programa de Posgrado *stricto sensu* en Enfermería (PPGEnf). En el período de recolección de datos, estaban matriculados 133 estudiantes, siendo 64 de maestría y 69 de doctorado. Se optó por el muestreo por conveniencia (no probabilístico) debido a la disponibilidad de los participantes. La recolección de datos ocurrió de mayo de 2023 a febrero de 2024.

### Criterios de selección

Se establecieron como criterios de inclusión: tener entre 18 y 59 años, debido a las particularidades relacionadas con el sueño en cada franja etaria y estar matriculado regularmente en el PPGEnf; como criterio de exclusión: estar en licencia o alejamiento de cualquier naturaleza durante el período de recolección de datos. Es oportuno aclarar que a los participantes no se les preguntó previamente a la entrega de los instrumentos de recolección de datos si utilizaban medicación con efectos sobre el sueño, apnea/hipopnea, trastornos neuropsiquiátricos o dolor crónico.

### Intervención educativa

La intervención educativa consistió en una sesión en línea en la que una de las autoras, estudiante de posgrado con experiencia en el tema y en recolección de datos cuantitativos, proporcionó orientaciones verbales, por medio de exposición dialogada, con auxilio de una presentación sobre aspectos generales, fases e importancia del sueño, ritmo circadiano y posibles perjuicios para la salud física, mental y emocional derivados de alteraciones en el sueño. También, sobre hábitos de higiene del sueño, presentando el concepto, comportamientos personales y factores ambientales que pueden ayudar a su mejora. Se invitó a los participantes a implementar esos hábitos en su rutina diaria.

El contenido abordado en la intervención educativa se basó en evidencias científicas identificadas en la revisión integrativa realizada previamente y en las directrices de la *Associação Brasileira do Sono* ^8^ ^-^ ^9^, adaptadas para la población de este estudio. Incluyeron orientaciones como ir a la cama, por la noche, cuando se esté somnoliento; mantener una rutina regular en el horario de acostarse y levantarse (independientemente de la hora a la que se haya dormido), incluso el fin de semana; evitar permanecer más de 30 minutos en la cama si se está despierto; si es necesario levantarse, evitar luz blanca o azul (encontrar un lugar para relajarse fuera del dormitorio y solo regresar a la cama con sueño, incluso durante la madrugada).

Evitar utilizar la cama para tareas que estimulen la actividad cerebral (leer, trabajar, ver TV, utilizar el teléfono móvil o cualquier pantalla que emita luminosidad); evitar actividades estresantes al acostarse, pudiendo anotar los compromisos en la agenda y reservar un período durante el día para concentrarse en los problemas que preocupan; evitar dormir siestas durante el día (en caso de ser necesario, limitar la duración a aproximadamente 30 minutos, después del almuerzo).

Restringir el uso de alcohol, nicotina y sustancias estimulantes (café, mate, energéticos) después de las 15h; evitar comer en exceso dentro de, aproximadamente, cuatro horas antes de acostarse; practicar ejercicio físico regularmente (hasta una hora antes del horario de dormir); tomar un baño tibio cerca de la hora de acostarse para relajarse; practicar ejercicios de control de la respiración en cualquier momento del día para relajarse; mantener el entorno donde se duerme oscuro, silencioso y con temperatura agradable.

Las sesiones en línea ocurrieron en diferentes turnos y se realizaron un total de 21 sesiones vía *Google Meet*. Cada participante fue asignado a un grupo para participar en la sesión, según la elección de la fecha y el horario previamente informados; se optó por la formación de grupos para permitir discusiones. En el turno de la mañana hubo cinco grupos, en el turno de la tarde 12 grupos y en el turno de la noche cuatro grupos. Los grupos tuvieron como mínimo 1 y como máximo 7 participantes y cada individuo participó una vez. A pesar del número variable de participantes en cada encuentro, la intervención siguió rigurosamente un guion previamente elaborado.

Cada encuentro tuvo una duración aproximada de 25-30 minutos. Al final de cada encuentro, se dispuso de aproximadamente 10 minutos para que los participantes compartieran sus experiencias, dificultades y facilidades relacionadas con sus rutinas y el sueño, así como para aclarar dudas sobre el tema.

Inmediatamente después de finalizar cada sesión en línea, se envió vía *WhatsApp* ^*®*^ a cada participante presente en el encuentro un folleto en *Portable Document Format* (PDF) y un video de 1 minuto y 20 segundos, ambos elaborados por los autores, con las orientaciones sobre higiene del sueño, posibilitando el acceso al contenido siempre que lo desearan.

Se enviaron cuatro mensajes de texto, uno por semana, incentivando y recordando los hábitos, brindando la oportunidad de aclarar posibles dudas y compartir la experiencia. El primer mensaje fue enviado siete días después de la participación en la intervención educativa; el segundo mensaje, siete días después del primero y así consecutivamente.

### Instrumentos

Se utilizó un cuestionario sociolaboral con variable cuantitativa relacionada con la edad, y variables cualitativas, categorizadas en sí o no, relacionadas con el sexo asignado al nacer, hijos, posgrado, desplazamiento hacia el posgrado y trabajo; cuestionario de hábitos de vida, con variables cualitativas, también categorizadas en sí o no, sobre el uso de medicamentos para dormir y síntomas de salud autoinformados; cuestionario sobre hábitos de sueño, con variables sobre duración, latencia, percepción sobre el sueño, somnolencia, hábitos de higiene del sueño y rutina de dormir.

Para verificar el grado de facilidad o dificultad para conciliar el sueño, se elaboraron preguntas en las que el participante marcaba la opción “fácil” o “difícil” para cuestiones como: “¿Cómo fue, para usted, por la noche, ir a la cama cuando estaba somnoliento? ¿Cómo fue, para usted, mantener una rutina regular en el horario de acostarse y levantarse?”. Estas preguntas fueron elaboradas por una estudiante de posgrado del curso de doctorado y analizadas por dos profesoras doctoras, con experiencia en investigaciones sobre la salud del trabajador y el sueño.

Para evaluar la calidad del sueño, se utilizó el Índice de Calidad del Sueño de Pittsburgh, versión validada en portugués de Brasil (PSQI-BR), con puntaje global que puede variar de 0 a 21 puntos, clasificando como calidad del sueño buena (≤ 4), calidad del sueño mala (5-10) y presencia de trastorno del sueño (≥ 11)^11^. Se optó por categorizar la variable en calidad del sueño “buena” (≤ 5) y “mala” (> 5 puntos), conforme utilizado en otro estudio^12^.

Para medir los hábitos de higiene del sueño, se utilizó el Índice de Higiene del Sueño, versión validada en portugués de Brasil (IHS-BR). Su puntuación puede variar de 13 a 65 puntos, siendo las puntuaciones más altas un indicativo de higiene del sueño inadecuada^13^. Para este estudio, se consideró higiene del sueño “media/buena” (≤ 34 puntos) y “mala” (≥ 35 puntos)^14^ ^-^ ^15^.

Se realizó una prueba piloto con los instrumentos de recolección de datos junto a los participantes de un grupo de investigación para verificar la claridad de las preguntas, el tiempo de diligenciamiento y posibles dificultades de interpretación. Tras ajustes en el formato, los instrumentos se consideraron adecuados para la recolección. 

### Recolección de datos

La recolección de datos ocurrió de forma en línea, en *Google Forms*, en tres etapas: antes, durante la implementación y después de la intervención. La intervención se ofrecía tras el diligenciamiento de los cuestionarios de la preintervención y consistía en la exposición dialogada y en el envío de cuatro mensajes de texto como refuerzo de los hábitos de higiene del sueño. Los cuestionarios de la posintervención se ponían a disposición una semana después del envío del cuarto y último mensaje.

Para una mejor visualización de las etapas del desarrollo del estudio, se presenta un diagrama de flujo en la Figura 1.

**Figura 1:** Diagrama de flujo del desarrollo de la investigación

### Análisis de los datos

Los datos recolectados fueron exportados desde *Google Forms*, organizados en el *software Microsoft Excel* ^*®*^ y analizados en el programa *Statistical Package for the Social Sciences* ^*®*^ (SPSS 25.0). Las variables categóricas se describen por la frecuencia absoluta (N) y relativa (%) y las cuantitativas por la media y la desviación estándar. La alteración entre los momentos, para cada hábito del sueño, calidad del sueño e índice de higiene del sueño, fue descrita y comparada antes y después de la intervención con uso de la prueba de chi-cuadrado de McNemar, considerándose para el rechazo de la hipótesis de nulidad un nivel de significancia de 5% (p<0,05).

### Aspectos éticos

Los aspectos éticos fueron respetados, conforme las recomendaciones de las Resoluciones 466/12 y 510/2016 del Consejo Nacional de Salud. El estudio fue aprobado por el Comité de Ética en Investigación de la *Universidade Federal de Santa Maria*, con Certificado de Presentación para Apreciación Ética n.º 66359622.6.0000.5346, bajo el dictamen n.º 5.847.306 de enero de 2023. Se menciona que en el Consentimiento Informado (CI) constaba la información sobre el uso de mensajes de texto vía *WhatsApp* ^*®*^ .

## Resultados

Respondieron los cuestionarios de la preintervención 53 estudiantes de posgrado en Enfermería (39,84% de la población); de estos, 11 no participaron en la intervención y dos no respondieron los cuestionarios de la posintervención. Completaron todas las fases del estudio 40 participantes, considerados para el análisis comparativo, que tenían una media de edad de 34 años (desviación estándar±8,11) (mínimo, 23; máximo, 54 años), el 95% (n=38) se asignaron el sexo femenino y 42,5% (n=17) tenían hijos. De los participantes, 50% (n=20) estaban cursando el doctorado, 25% (n=10) necesitaban desplazarse a otra ciudad o estado para realizar el posgrado, 87,5% (n=35) trabajaban, 37,5% (n=15) estaban siguiendo algún tratamiento de salud y 20% (n=8) utilizaban medicación para dormir.

Se realizaron análisis antes y después de la intervención (n=40), en los cuales se compararon los hábitos de sueño, la calidad del sueño (PSQI-BR) y la higiene del sueño (IHS-BR) de los participantes.

A continuación, la Tabla 1 presenta la comparación antes y después de la intervención entre variables relacionadas con los hábitos de sueño de los estudiantes de posgrado.

**Tabla 1:** Variables relacionadas con los hábitos de sueño antes y después de la intervención con estudiantes de posgrado (n = 40). Santa Maria, RS, Brasil, 2023-2024

Variable	Antes de la intervención (%)	Después de la intervención (%)	p-valor*
Considera suficientes las horas de sueño			
Sí	16 (40,0)	24 (60,0)	0,057
No	24 (60,0)	16 (40,0)	
Dificultad para mantener el sueño			
Sí	15 (37,5)	9 (22,5)	0,146
No	25 (62,5)	31 (77,5)	
Dificultad para iniciar el sueño			
Sí	13 (32,5)	8 (20,0)	0,125
No	27 (67,5)	32 (80,0)	
Dificultad para despertar			
Sí	23 (57,5)	16 (40,0)	0,065
No	17 (42,5)	24 (60,0)	
Somnolencia durante el día			
Sí	26 (65,0)	22 (55,0)	0,388
No	14 (35,0)	18 (45,0)	
Total	40 (100)	40 (100)	

*Prueba de chi-cuadrado de McNemar, significativo si valor p < 0,05

No se identificó significancia estadística en esos hábitos de sueño. A continuación, en la Figura 2, se puede visualizar una diferencia estadística entre la cantidad de horas de sueño, la percepción sobre el sueño, la sensación de cansancio y poseer hábitos de higiene del sueño antes y después de la intervención.

**Figura 2:** Hábitos de sueño antes y después de la intervención con estudiantes de posgrado (n = 40). Santa Maria, RS, Brasil, 2023-2024

*Prueba de chi-cuadrado de McNemar, significativo si valor p < 0,05

Tras la intervención, hubo un aumento porcentual de participantes que elevaron sus horas de sueño de < 7h a ≥ 7h a 9h por noche (p=0,049) (22,5%), que percibieron mejora sobre su sueño (p=0,016) (17,5%) y que adquirieron hábitos de higiene del sueño (p<0,001) (67,5%). Se observó una reducción porcentual de estudiantes que se despertaban sintiéndose cansados (p=0,039) (20,2%).

La Figura 3 muestra la calidad del sueño, según el PSQI-BR, antes y después de la intervención.

**Figura 3:** Calidad del sueño antes y después de la intervención con estudiantes de posgrado (n = 40). Santa Maria, RS, Brasil, 2023-2024

*Prueba de chi-cuadrado de McNemar, significativo si valor p < 0,05

La calidad del sueño no presentó significancia estadística (p=0,774) después de la intervención. A continuación, la Figura 4 muestra la higiene del sueño, de acuerdo con el IHS-BR, antes y después de la intervención.

**Figura 4:** Higiene del sueño antes y después de la intervención con estudiantes de posgrado (n = 40). Santa Maria, RS, Brasil, 2023-2024

*Prueba de chi-cuadrado de McNemar, significativo si valor p < 0,05

La higiene del sueño presentó mejora estadísticamente significativa (p=0,012) después de la intervención, es decir, los participantes fortalecieron las prácticas y hábitos que ayudaron a un patrón de sueño más saludable, pues recibieron orientación para dormir bien. Se menciona que hubo un aumento en el número de participantes que presentaron higiene del sueño media/buena después de la intervención.

Con respecto a los datos descriptivos sobre los síntomas autoinformados antes y también después de la intervención (n=40), los participantes pudieron marcar una o más opciones. Después de la intervención, hubo reducción en los porcentajes de los síntomas: dificultad de concentración y/o memorización, de 77,5% (n=31) a 22,5% (n=9); dolor de cabeza, de 57,5% (n=23) a 35% (n=14); disminución o aumento del apetito, de 50% (n=20) a 5% (n=2); irritabilidad, de 50% (n=20) a 27,5% (n=11); sensación de mala digestión o acidez, de 40% (n=16) a 7,5% (n=3); somnolencia diurna excesiva, de 40% (n=16) a 32,5% (n=13) y sensación de infelicidad, de 25% (n=10) a 12,5% (n=5).

Los resultados descriptivos respecto al grado de facilidad o dificultad que los participantes observaron en la implementación de cada hábito de higiene del sueño señalaron que mantener el cuarto oscuro, silencioso y con temperatura agradable para dormir fue observado cómo de fácil implementación en la rutina por 95% (n=38) de los participantes, mientras que evitar la cama para realizar tareas que estimulen la actividad cerebral fue considerado lo más difícil de implementar, abarcando 37,5% (n=15) de los participantes.

A partir del análisis de los datos, se identificó que 100% (n=40) comprendieron qué es la higiene del sueño y cuáles son los hábitos; 87,5% (n=35) percibieron mejoría en la rutina/hábitos de higiene del sueño tras la intervención y el mismo porcentaje identificó que la intervención ayudó de alguna forma a la mejora del sueño. Además, 90% (n=36) señalaron que el refuerzo, por medio de mensajes de texto, ayudó en la adhesión a la práctica.

Del total, 75% (n=30) reportaron que hubo factores que interfirieron o dificultaron sus hábitos de sueño, como situaciones familiares, cuestiones laborales o académicas específicas durante el desarrollo del estudio.

## Discusión

La intervención educativa no alteró significativamente la calidad del sueño. Mejorar hábitos es un proceso esencial para dormir mejor, pero no siempre garantiza una buena calidad del sueño^10^. Esto puede explicarse por el hecho de que el sueño está influido por una combinación de factores genéticos, fisiológicos, psicológicos, emocionales, ambientales y condiciones de salud^7^ ^,^ ^16^ ^-^ ^17^.

Asimismo, se menciona que el consumo excesivo de cafeína, alcohol, el uso inadecuado de dispositivos electrónicos, el sedentarismo, la propia privación del descanso en detrimento del cumplimiento de actividades, plazos, producción académica, compromisos laborales y personales pueden perjudicar el sueño y estar asociados a preocupación, ansiedad y estrés^1^ ^,^ ^4^ ^,^ ^18^. Esto puede observarse en los resultados, pues un mayor número de estudiantes informó que hubo factores que interfirieron o dificultaron el sueño.

Estos factores pueden crear un ciclo en el cual el sueño es frecuentemente desatendido, lo que hace imprescindible la atención a la calidad del sueño de los estudiantes de posgrado en enfermería. El descanso inadecuado, además de perjudicar la salud física y mental, puede comprometer la capacidad de estos estudiantes para desempeñar sus funciones de forma segura y eficaz, especialmente cuando actúan en entornos de alta responsabilidad, como instituciones de salud. Esto puede llevar a errores graves, ya que la fatiga impacta directamente en la atención y en la toma de decisiones^3^.

Además, estos individuos enfrentaban una serie de síntomas relacionados con la privación del sueño. Esto indica que el reposo inadecuado puede afectar directamente la salud física y emocional^12^, lo que preocupa al tener en cuenta la media de edad de 34 años, indicando la necesidad de intervenciones educativas sobre el sueño para promover una perspectiva de futuro con hábitos más saludables.

Sin embargo, tras la intervención, la higiene del sueño de los estudiantes de posgrado presentó mejora estadísticamente significativa. Esto sugiere relación con el aumento del conocimiento de los participantes sobre hábitos saludables, como se encontró en otra investigación^2^, lo que puede ofrecerse por medio de la intervención educativa realizada.

La falta de conocimiento sobre aspectos fisiológicos del sueño y las consecuencias de su privación, dado que no son temas abordados durante la formación académica y profesional en general^19^, puede favorecer malos hábitos, lo que está ligado a daños a la salud. Así, las intervenciones educativas se vuelven una forma de instrucción relativamente accesible e indispensable.

Más allá del conocimiento, a partir de los resultados se sugiere la implementación práctica de los hábitos en la rutina de los estudiantes de posgrado. Se identificó un aumento expresivo en el número de individuos que adquirieron hábitos de higiene del sueño tras la intervención, siendo una estrategia factible y satisfactoria para presentar y discutir el tema, viabilizando la aplicación de hábitos saludables en lo cotidiano a partir del conocimiento adquirido.

Se destaca que la implementación de los hábitos considera las particularidades de cada individuo, siendo un proceso continuo. Aplicar los hábitos de higiene del sueño, de forma práctica y constante, es fundamental para garantizar efectos positivos a largo plazo. La mejora del sueño y de la salud está directamente ligada a esos hábitos, lo que hace esencial que sean considerados un compromiso permanente y no una solución temporal a los problemas de sueño. Así, se ayudará en la prevención de daños y agravios a la salud, promoviendo calidad de vida y bienestar^2^. 

Corroborando los hallazgos del presente estudio, una investigación identificó que 90,3% de los participantes duermen en un ambiente oscuro y silencioso, mientras que 83,5% utilizaban el celular acostados en la cama, siendo un hábito desfavorable para el sueño^20^. La discusión acerca del uso de dispositivos electrónicos antes de dormir por parte de estudiantes de posgrado merece atención.

Una investigación señaló que el uso del *smartphone* en la cama tiene efectos adversos significativos en la latencia del sueño, en el tiempo de vigilia y en la frecuencia cardíaca de adultos. Esto se debe a la emisión de luz azul, que interfiere en la producción de melatonina y en el ritmo circadiano por medio de células ganglionares de la retina (RGC) que contienen melanopsina (receptor que mide la intensidad de la luz recibida)^18^. Al investigar el efecto de la reducción de la luz azul de la pantalla del *smartphone*, durante la noche, en adultos jóvenes, una investigación identificó que hubo mejora en la calidad subjetiva del sueño, en el funcionamiento diurno y en la hora de acostarse^21^.

Un estudio evaluó el impacto de la aplicación del filtro de luz azul en la calidad del sueño, pero no encontró efectos positivos sostenidos en esos parámetros^22^; otro no encontró mejoras en el sueño y en la salud mental en adultos jóvenes tras intervenir con alteraciones automatizadas en la temperatura de color de la pantalla de dispositivos electrónicos^23^, es decir, el impacto de la filtración de luz azul permanece discutible. Así, se vuelve imprescindible la orientación sobre evitar el uso de pantallas antes de dormir.

Entre algunas estrategias para restringir la exposición excesiva, es primordial tener el hábito de alejarse conscientemente de los aparatos digitales. Se pueden establecer límites de tiempo para el uso de *smartphone*, computadora, *notebook*, tableta y televisión, existiendo funciones, configuraciones y aplicaciones disponibles para auxiliar en ese control. Determinar horarios específicos para el uso, preferentemente durante el día, absteniéndose de mantenerse conectado por la noche y creando horarios designados “*offline*”^24^.

Evitar el uso automático, que ocurre cuando no se tiene un objetivo específico para utilizar el dispositivo; aliado a ello, desactivar y/o gestionar notificaciones^24^. Asimismo, producir espacios placenteros y sin pantallas, buscando realizar actividades al aire libre, en presencia de familiares y amigos, practicando hobbies, realizando actividades recreativas, ejercicios físicos y/o lecturas. Recordando que esas estrategias dependen principalmente del interés, la disposición, la organización y los límites del individuo que busca el autocuidado.

La gestión de las pantallas antes de dormir entra en el contexto de prepararse para el momento de reposo, lo que incluye adecuar y adaptar la habitación donde se duerme, favoreciendo el descanso. Un estudio refuerza el hecho de mantener el cuarto oscuro, silencioso y con temperatura templada, ayudando en la duración del sueño^17^. Es una práctica relativamente fácil y sin costo, que puede orientarse, presentando resultados positivos.

Tras la intervención, hubo mejora significativa en las horas y en la percepción subjetiva sobre el sueño. Esto retrata que las medidas objetivas y la percepción de los participantes convergen. Esta mejora es relevante, dado que, en general, se necesitan de 7 a 9h de sueño por noche para adultos. Cabe resaltar que hay particularidades, pues algunos adultos se sienten bien con 6 h de sueño, mientras que otros necesitan 10h^9^.

Es importante que cada individuo identifique y respete sus necesidades y límites, priorizando mantener la cantidad de horas indispensables para un sueño adecuado. Ese resultado puede estar vinculado a la mejora significativa en la percepción sobre el cansancio tras la intervención. La cantidad de sueño adecuada, resultante de una rutina más saludable, incide positivamente en el desempeño y la disposición.

El refuerzo, por medio de mensajes de texto, y el material educativo puesto a disposición con el contenido abordado facilitó el acceso a la información y estimuló la práctica. Los videos educativos son ampliamente utilizados para la educación en salud de la población, pudiendo traer beneficios para la salud y cambios conductuales^25^.

De la misma forma, un estudio que tuvo por objetivo describir la elaboración y validación de una cartilla educativa para enfermeros identificó que el material distribuido de forma en línea e impresa representó un medio de contribución y fortalecimiento de la práctica. La diagramación del contenido en textos, tópicos e ilustraciones impulsó la utilización del material, facilitando la comprensión^26^.

La opción por el estudio cuasi experimental se debió a la aplicabilidad, al perfil de los participantes y a ser una intervención comunitaria. Los sesgos de este estudio involucraron la no aleatorización de los participantes, en la que los grupos pueden diferir en características que no fueron controladas; hubo la ausencia de control de factores externos, como la ocurrencia de situaciones personales, académicas o laborales que podrían interferir en el sueño, correlacionados tanto con el tratamiento como con el desenlace, pudiendo distorsionar la estimación del efecto causal.

Las limitaciones se refieren al tipo de estudio, que puede sugerir relaciones causales, pero la falta de aleatorización y el control de factores externos impiden una conclusión definitiva. También al hecho de que el estudio se realizó en un único PPGEnf del estado de Rio Grande do Sul y, además, a la pérdida de participantes durante el desarrollo de la investigación. El tamaño de la muestra y la dificultad para eliminar sesgos y factores de confusión pueden limitar la generalización de los resultados.

Este estudio contribuye al avance del conocimiento científico en el área de la salud y la enfermería, pues presenta una intervención educativa sobre higiene del sueño como una práctica abarcadora y de bajo costo, aplicable a diversos contextos. Sensibilizar a la población sobre la importancia de hábitos saludables colabora con la prevención y la promoción del sueño, la salud física y mental.

## Conclusión

La intervención educativa mejoró la higiene del sueño y la percepción subjetiva sobre el sueño de estudiantes del posgrado *stricto sensu* en Enfermería. Tras la intervención, hubo un aumento significativo en el número de estudiantes que adquirieron hábitos de higiene del sueño, con incremento en la cantidad de horas de sueño y mejora en la percepción sobre el cansancio. No hubo mejora significativa en la calidad del sueño de los estudiantes de posgrado, después de la intervención.

## Declaración de Disponibilidad de Datos:

Todos los datos generados o analizados durante este estudio están incluidos en este artículo publicado.
